# Disentangling the absorption lineshape of methylene blue for nanocavity strong coupling

**DOI:** 10.1515/nanoph-2025-0474

**Published:** 2025-11-25

**Authors:** Santiago A. Gomez, Emmi K. Pohjolainen, Dmitry Morozov, Ville Tiainen, J. Jussi Toppari, Gerrit Groenhof

**Affiliations:** Nanoscience Center and Department of Chemistry, University of Jyväskylä, P.O. Box 35, 40014 Jyväskylä, Finland; Nanoscience Center and Department of Physics, University of Jyväskylä, P.O. Box 35, 40014 Jyväskylä, Finland

**Keywords:** absorption spectra, vibronic progression, plasmonic nanocavity, strong coupling, cucurbituril, methylene blue

## Abstract

Cucurbit[7]uril molecules form non-covalent host – guest complexes with small molecular dyes. In addition, cucurbit[7]uril can also bind gold nanoparticles on gold surfaces with a 0.9 nm gap, creating plasmonic nanocavities for the dyes, with extreme confinement of the electromagnetic field. For methylene blue in such cavities, single molecule strong coupling was inferred from a complete disappearance of a characteristic shoulder in its spectrum, attributed to dimer removal. Yet, the shoulder’s origin remains debated. Using atomistic simulations, we show that it arises from both dimerization and vibronic progression. While cucurbit[7]uril binding removes the dimer contribution, vibronic progression persists. As this conflicts with previous reports, we also measured the spectra. In line with our computations, the shoulder remains visible when cucurbit[7]uril binds methylene blue. These results clarify the spectral features and pave the way for atomistic models of single-molecule strong coupling in nanoparticle-on-mirror cavities.

## 1 Introduction

Polaritonic chemistry is a new field that aims at manipulating the chemical properties of materials with optical resonators [1], [2], [3]. Because of the enhanced light–matter interaction within such resonators, excitations of the material can hybridize with the confined light modes of the optical resonator to form polaritons [4]. Although changes in material properties have been attributed to the formation of such polaritons [1], there is as yet no consensus on how polariton formation can lead to such changes [5], [6], [7], [8], [9], [10].

For organic materials, this so-called *strong coupling* regime is typically achieved by placing very large numbers of molecules either inside a Fabry–Pérot microcavity [11], or on top of a metal surface [12], [13], of a plasmonic lattice [14], or of a distributed Bragg mirror [15], [16]. However, due to challenges associated with the description of large numbers of molecules, theoretical efforts have focused predominantly on developing quantum chemistry approaches for single molecule strong coupling [17], [18], [19], [20]. Because of the large discrepancy in the number of molecules, results obtained with such models on single-molecule coupling cannot be directly compared to experiments in the collective strong coupling regime. Instead, experiments on single-molecule strong coupling are urgently needed to verify the validity of these single-molecule theories. Reaching the strong coupling regime with only a single molecule under ambient conditions in such experiments requires sub-wavelength confinement of the electromagnetic field, which can only be achieved with nanoplasmonic resonators [21], [22], [23], [24], [25], [26], [27].

**Figure 1:**
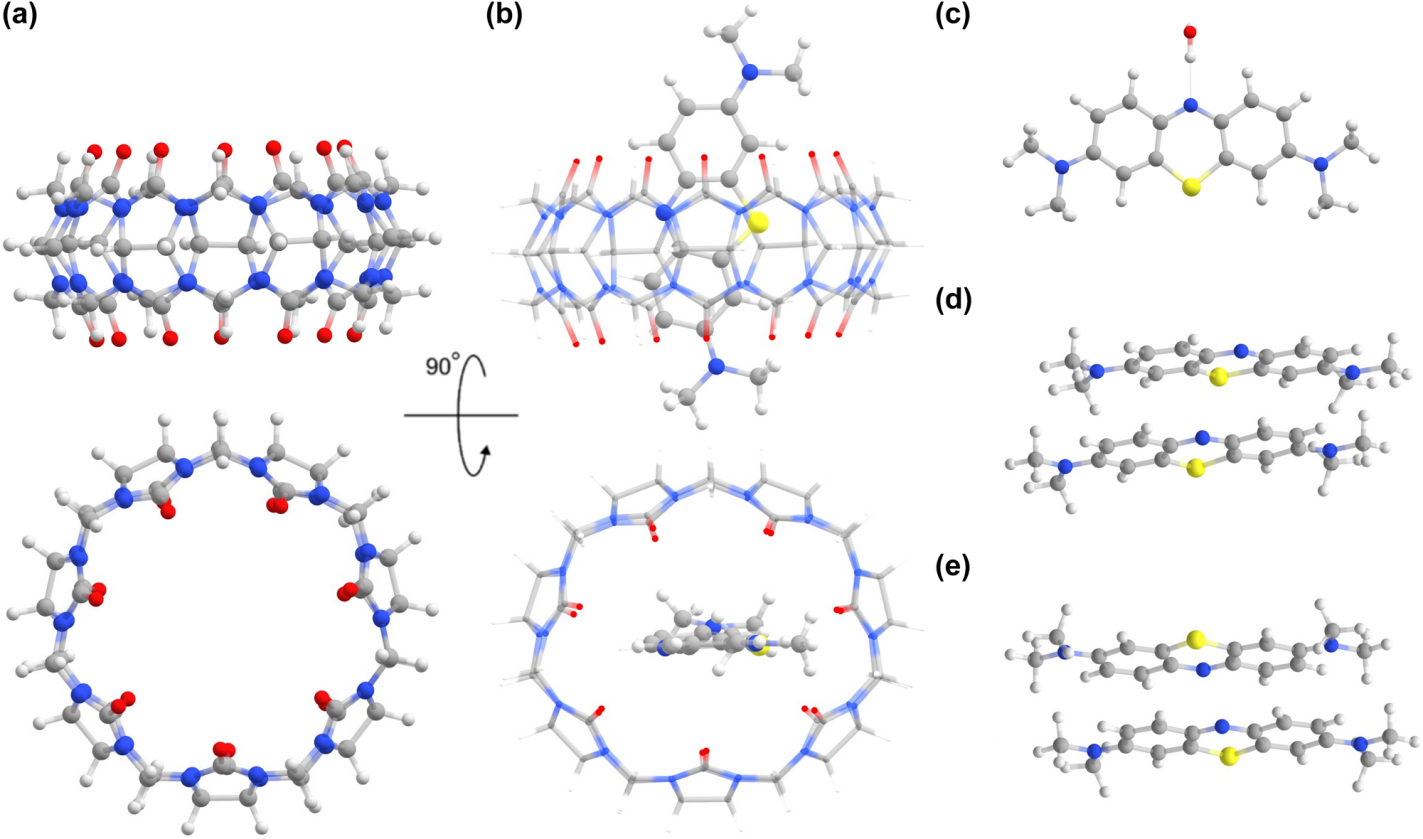
Side (**top**) and front (**bottom**) views of a cucurbit[7]uril molecule without (a) and with (b) methylene blue. A methylene blue monomer with an explicit water molecule bound to the nitrogen atom of the central ring, is shown in panel (c). Panels (d) and (e) show the parallel and antiparallel dimer conformations. Carbon atoms are shown in gray, oxygen atoms in red, nitrogen in blue, sulfur in yellow and hydrogen in white.

The first demonstration of single-molecule strong coupling was reported in 2016 by Baumberg and co-workers for a methylene blue (MeB) dye in a nanoparticle on mirror (NPoM) plasmonic nano-cavity [24]. The NPoM nano-cavity was formed via self-assembly of a gold nanoparticle on top of a gold surface in the presence of cucurbit[7]uril (CB7). As illustrated in Figure 1(a), CB7 is a barrel-shaped organic molecule with a central cavity in which small molecules, such as the MeB dye, can bind. In addition, the carbonyl oxygen atoms on both ends of the barrel can form strong bonds with gold atoms and hence bind the gold nanoparticle onto the surface with a gap of 0.9 nm [28]. The plasmonic mode originating from the interaction between the nanoparticle and its mirror image, is thus extremely confined within this gap and can therefore strongly couple with the electronic excitation of a methylene blue dye inside the CB7 (Figure 1(b)). Indeed, in the experiments, Rabi splitting was observed when the plasmonic mode was resonant with the methylene blue absorption [24].

The key to concluding that single-molecule strong coupling had been achieved, was the assumption that the cucurbit[7]urils can bind at most one methylene blue molecule. To validate this assumption, also the absorption spectrum of methylene blue in water was measured, both with and without cucurbit[7]uril in a 1:10 MeB:CB7 ratio. Without cucurbit[7]uril, the absorption spectrum of methylene blue had a maximum at 678 nm (1.83 eV) with a shoulder at 632 nm (1.96 eV), both of which appeared red-shifted with respect to previous measurements in water, for which an absorption maximum was reported at 664 nm (1.87 eV) and a shoulder at 612 nm (2.03 eV) [29], [30], [31], [32], [33]. With cucurbit[7]uril, the shoulder disappeared, while the main absorption peak blue-shifted by 14 nm (39 meV) to 664 nm. Because the shoulder was attributed to the methylene blue dimer [30], [34], its disappearance in the presence of cucurbit[7]uril was taken as direct evidence that there is at most a single methylene blue guest inside a cucurbit[7]uril host. However, the nature of the shoulder in the methylene blue spectrum is contested, and has not only been attributed to the dimer [30], [34], [35], but also to vibronic progression [32], [36], [37], [38], to a higher-energy electronic state [39], [40], to mesomerism [41], and to trimers [33]. Because of these controversies, taking the absence of the shoulder, or the blue shift, as direct evidence that only a single methylene blue can form a host-guest complex with cucurbit[7]uril, may require some caution.

Because the NPoM is currently the only platform available for reaching the single-molecule strong coupling regime under ambient conditions, it is an important experimental reference system for theory. Therefore, understanding the molecular details of this system, and in particular how binding of the dye in the cucurbit[7]uril nano-cavity affects its optical properties, is essential for testing theoretical models. Here, we therefore address the controversies regarding the nature of the shoulder in the methylene blue absorption spectrum and the influence of cucurbit[7]uril on that spectrum. Using a range of computational techniques in combination with optical absorption spectroscopy, we could resolve discrepancies between previously published spectra, and validate an atomistic host-guest model for future molecular dynamics simulations of the complete NPoM system.

## 2 Materials and methods

### 2.1 Computations

#### 2.1.1 Structures

The methylene blue starting structure was built with Avogadro [42]. The two dimers were obtained from 20 ns molecular dynamics (MD) simulation of two MeB molecules in water using GROMACS 2022.3 [43] with the CHARMM36 force field [44]. All frames of the MD trajectory were clustered with a 0.188 nm RMSD cutoff. The two most populated clusters correspond to the parallel and antiparallel dimers shown in Figure 1(d) and (e). The initial structure for the MeB – CB7 complex was created with the ASE python package [45] by aligning the center of mass of MeB with that of CB7, followed by translating MeB in vertical direction with respect to CB7 until one end of the MeB was at the bottom of the CB7 barrel. The same approach was used to obtain the initial structures of the parallel and antiparallel MeB dimers in CB7 (MeB_2_–CB7).

#### 2.1.2 Quantum chemistry calculations

Vibronic spectra were computed with FCClasses version 3.0 [46] based on harmonic vibrational normal modes obtained with Gaussian16 [47] at the CAMB3LYP/6-31G(d) level of time-dependent density functional theory (TDDFT) [48], [49], [50], [51] including Grimme’s D3 dispersion correction [52] and an implicit representation of the water based on the polarizable continuum model (*ϵ*_*r*_ = 80) of Tomasi and co-workers [53], [54]. Due to the large difference between the electron density in the ground and excited states, which the adiabatic approximation employed in the CAM-B3LYP or any other functional, cannot capture accurately [55], [56], [57], the TDDFT excitation energies are significantly overestimated, in line with previous calculations [38], [40]. To account for these shortcomings of the TDDFT method, we also computed the excitation energies for all systems at the CIS(D)/6-31G(d) level of *ab initio* theory [58]. At this level, we find a vertical excitation energy of 2.34 eV for Methylene Blue in vacuum. As shown in the Supporting Information (SI, Figure S9), the topologies of the accessible regions of the excited state potential energy surfaces of methylene blue are not strongly affected, and we can thus use the difference between the TD-CAMB3LYP/6-31G(d) and CIS(D)/6-31G(d) electronic energies as an offset to compensate for the overestimation of the excitation energies at the TDDFT level, at least qualitatively. All vibronic absorption spectra were computed within the Adiabatic Hessian approximation with the time independent approach [59] at a temperature of 300 K. The calculated excitations were convolved with Gaussian functions of 0.022 eV width. Further details of these calculations, including a concise description of the approximations and their validity for the systems at hand, are provided in the SI.

#### 2.1.3 Classical molecular dynamics simulations

The force field parameters (CHARMM36 FF [44]) for MeB and CB7 were obtained with ligand reader and modeler [60] in CHARMM-GUI [61]. Both MeB–CB7 and MeB_2_ – CB7 complexes were relaxed with classical MD simulations of 100 ns in explicit water (TIP3P [62]) at constant pressure and temperature using the c-rescale barostat (*p*_ref_ = 1 bar; *τ*_*p*_ = 2 ps) [63] and v-rescale thermostat (*T*_ref_ = 300 K; *τ*_*T*_ = 1 ps) [64], respectively. All classical MD simulations were performed with the GROMACS-2023.3 software package [43].

### 2.2 Absorbance measurements

#### 2.2.1 Materials

Absorbance measurements were performed using a PerkinElmer Lambda 650 UV–Vis spectrometer. Methylene blue (MeB, CAS: 61-73-4) and Cucurbit[7]uril (CB7, CAS: 259886-50-5), were purchased from MedChemExpress (Bio-Mediator on Elektrokem Oy, Finland). Measurements were conducted in disposable UV micro cuvettes (Brand GmbH, Germany) with outer size of 12.5 × 12.5 × 45 mm^3^ and with a reduced light path of 4.5 mm.

#### 2.2.2 Samples

Stock solutions of MeB and CB7, as well as test solutions containing MeB alone and MeB mixed with CB7 at different molar ratios (Table S4, SI), were prepared using Millipore-grade water, which was also used as the reference for background subtraction. The initial MeB concentration in all test solutions was 400.5 μM (4.005 ⋅ 10^−4^ M). After measuring the undiluted test solutions, they were diluted in successive iterations by transferring 794 μL of the previously measured solution into 206 μL of Millipore water.

#### 2.2.3 Absorption spectra

Absorption spectra were recorded over the wavelength range 400–800 nm, at 1 nm intervals, using an exposure time of 0.04 s and a single accumulation. The monochromator slit width was set to 2 nm. The ambient laboratory temperature during all measurements was maintained at 21.8 °C.

Small baseline variations were observed between spectra, which we attribute to minor differences in the optical path lengths of the disposable cuvettes used for the test solutions. While the same reference cuvette was used throughout the experiment, the test cuvettes were replaced between measurements, causing minor baseline shifts likely due to differences in wall thickness or surface imperfections. To correct for these shifts without altering the spectral shape, a simple linear baseline correction was applied by subtracting the minimum absorbance value from each spectrum, and aligning the lowest point to zero. This approach preserves the relative spectral features and improves comparability across measurements.

## 3 Results and discussion

### 3.1 Absorption spectra of methylene blue in water

**Figure 2:**
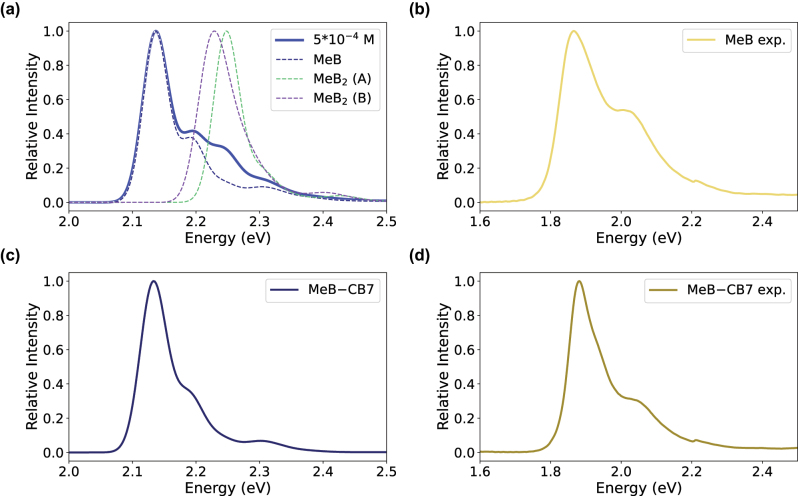
Computed and measured absorption spectra. (a) Simulated absorption spectra of methylene blue monomer (dashed blue) and dimers in the parallel (A) and antiparallel (B) stacking conformations. The total absorption spectrum of MeB solution at 5 ⋅ 10^−4^ M obtained as a superposition of monomer and dimer spectra with eq. (1), is also shown (solid blue). (b) Experimental absorption spectra for a solution of MeB at 5 ⋅ 10^−5^ M. (c) Simulated absorption spectra for methylene blue monomer in complex with cucurbit[7]uril and (d) experimental absorption spectra for a 1:10 solution of MeB (5 ⋅ 10^−5^ M) and CB7 (5 ⋅ 10^−4^ M). The minor discontinuities around 2.2 eV in panels b and d arise from automatic long-pass filter switching in the spectrometer light path and are not intrinsic to the sample.

First, we focused on the nature of the shoulder of MeB in water and computed the absorption spectra of the monomer and dimer, which are the dominant species at low concentrations [30], [34]. For the monomer, the spectrum shown in Figure 2(a), consists of a main peak at 2.14 eV and a shoulder around 2.19 eV, which become 1.83 eV (677 nm) and 1.88 eV (659 nm), respectively, after the *ad hoc* CIS(D) correction of the TD-CAMB3LYP excitation energies. In line with previous calculations [37], [38], the main peak is dominated by the 0 → 0 transition (*i.e.*, from the vibrational ground state ( $v_{j}^{{\text{S}}_{0}}=0$ ) of all normal modes on the electronic ground state (S_0_) potential energy surface into the vibrational ground state of all modes on the electronic excited state (S_1_) potential energy surface ( $v_{j}^{{\text{S}}_{1}}=0$ ), as illustrated in Figure S1 of SI). The shoulder originates from a combination of $v_{j}^{{\text{S}}_{0}}=0\to v_{j}^{{\text{S}}_{1}}=1$ transitions in normal modes *j* = 25 and *j* = 27 (*i.e.*, excitation into the first vibrational excited state of these modes on the S_1_ potential energy surface), with harmonic frequencies of 454 and 510 cm^−1^, respectively. These, and other normal modes contributing to the spectrum, are visualized in Figure S3 (SI). The shape of the calculated spectrum matches well with the corresponding experimental absorption spectrum of a solution of MeB shown in Figure 2(b) (discussed further below).

For the dimer, we optimized two conformations with a parallel (A, Figure 1(d)) and antiparallel (B, Figure 1(e)) stacking of the monomers [65]. Because the energy difference between the two dimer conformations is within one kcal mol^−1^ (Table S1, SI), we simulated the vibronic spectra for both. The calculated absorption spectra, shown in Figure 2(a), have an absorption maximum at 2.25 eV and 2.23 eV for the parallel and antiparallel dimer conformation, respectively. In both conformations, the bright state corresponds to the upper exciton, whereas the lower exciton state is dark, indicating that the dimers are H-aggregates [66], in line with previous results [34]. After CIS(D) correction, the two dimer maxima are at 1.93 eV and 1.91 eV and thus very near the shoulder of the monomer absorption spectrum at 1.88 eV.

Next, we computed the total spectrum for an aqueous solution of MeB at initial concentration of *C*_*i*_ = 5 ⋅ 10^−4^ M. At this concentration, both monomeric and dimeric species should be present. The total absorptivity of the solution was therefore calculated as a linear combination of the monomer and dimer spectra:

(1) $$\begin{array}{cc} A\left(\nu \right)= & \varepsilon_{\text{M}}\left(\frac{-1+\sqrt{1+8K_{\text{D}}C_{i}}}{4K_{\text{D}}}\right)A_{\text{M}}\left(\nu \right)\\ & \;+\varepsilon_{\text{D}}\left(\frac{C_{i}}{2}-\frac{-1+\sqrt{1+8K_{\text{D}}C_{i}}}{8K_{\text{D}}}\right)A_{{\text{D}}_{\text{A}}}\left(\nu \right)\\ & \;+\varepsilon_{\text{D}}\left(\frac{C_{i}}{2}-\frac{-1+\sqrt{1+8K_{\text{D}}C_{i}}}{8K_{\text{D}}}\right)A_{{\text{D}}_{\text{B}}}\left(\nu \right) \end{array}$$

where *A*_M_(*ν*), $A_{{\text{D}}_{\text{A}}}\left(\nu \right)$ and $A_{{\text{D}}_{\text{B}}}\left(\nu \right)$ are the computed absorption of the monomer and of the two dimer conformations, respectively, as a function of excitation energy (*ν*). The constant values *ɛ*_M_ and *ɛ*_D_ are the molar absorptivity of the monomer and the dimers, and *K*_D_ is the dimerization constant reported by Florence and Naorem [35]. The resulting spectrum, plotted in Figure 2(a), shows the effect of the dimers on the shape of the shoulder. Although the vibronic shoulder of the monomer and main absorption peaks of the dimers do not align perfectly, we note that the deviation is well within the expected uncertainty of TDDFT predictions (0.2–0.3 eV) [67]. Indeed, shifting the dimer spectra by as little as 0.02 eV significantly increases the agreement between the calculated (Figure S5) and experimental line shapes (Figure S6(c)). Absorption spectra calculated for other concentrations are shown in Figure S6(a) and (b) of SI, and are in line with experiment (Figure S6(c)). Based on these findings, we attribute the shoulder observed in the solution spectrum of MeB, to a combination of monomeric vibronic progression and dimeric excitons [32], [34].

In contrast, Dunnet *et al.*, attributed the shoulder to intensity borrowing due to strong non-adiabatic coupling between the first (S_1_) and second (S_2_) singlet excited states of the monomer based on hybrid quantum mechanics–molecular mechanics (QM/MM) molecular dynamics (MD) simulations [40]. To investigate this possibility, we monitored the S_1_–S_2_ energy gap along a classical QM/MM MD trajectory, in which the chromophore was described at the CAM-B3LYP/6-31G(d) level of theory, while the water was modeled with the TIP3P point-charge model [62]. As shown in Figure S10, the S_1_ and S_2_ states remain energetically well-separated, with an average gap of 0.36 eV, which is somewhat larger than the average energy gaps reported by Dunnett *et al.* for various DFT/MM models (0.07–0.23 eV) [40]. At the CIS(D)/6-31G(d)//TIP3P level of theory the gap is 0.67 eV on average. Because the non-adiabatic coupling that determines the efficiency for intensity borrowing, is inversely proportional to the energy gap between the electronic states [68], we speculate that a gap of 0.67 eV would be too large for a sufficiently fast population transfer from S_1_ into S_2_ and, therefore, rule out this mechanism as the main origin of the shoulder.

### 3.2 Absorption spectra of cucurbit[7]uril – methylene blue host-guest complex

To understand how binding to cucurbit[7]uril influences the methylene blue absorption, we next computed the vibronic spectra of the cucurbit[7]uril – methylene blue host-guest complex in (implicit) water. At the CAM-B3LYP/6-31G(d)//PCM level, the complexation energy, corrected for the basis set superposition error, is 34 kcal/mol (Table S2, SI). In addition, we estimated a complexation free energy of 14.5 kcal/mol from enhanced sampling molecular dynamics simulations in explicit water at 300 K, using the CHARMM36 force field [44], in combination with the accelerated weight histogram (AWH) method (Figure S12, SI) [69]. Both calculations support strong binding of MeB to CB7.

As shown in Figure 2(c), the lineshape of the calculated absorption spectrum of the MeB–CB7 complex in water closely matches the spectrum of methylene blue in solution with a main absorption band at 2.13 eV and a shoulder at 2.18 eV. The shoulder is due to vibronic progression that involves the same vibrational modes as in solution (see Figure S4 and Table S3 in SI), but with slightly blue-shifted frequencies. These results thus suggest that although binding a single methylene blue to the cucurbit[7]uril removes the contribution of the dimer, the absorption spectrum still features a shoulder. This prediction is in contrast to the complete disappearance of this shoulder in the spectra of Chikkaraddy *et al.* [24].

To resolve this discrepancy and verify the validity of our predictions, we measured the absorption spectrum of methylene blue in water at various concentrations both with and without cucurbit[7]uril. A spectrum measured at the same 1:10 MeB:CB7 ratio as Chikkaraddy *et al.* [24], is shown in Figure 2(d). A comparison between the measured and computed spectra reveals that despite the systematic blue shift of the calculated absorption, which can be attributed to shortcomings of (*i*) the DFT functional, (*ii*) the implicit water model, and (*iii*) the small basis set, the absorption lineshapes are in good agreement. Specifically, the absorption spectra retain the shoulder in the presence of CB7, even at the lowest MeB concentrations (Figure S6(c), SI). The reduced intensity of this shoulder furthermore suggests that the formation of a MeB–CB7 host-guest complex is thermodynamically more favorable than MeB dimerization, at least at the 1:10 MeB:CB7 ratio (Figure S13, SI).

In our experiments, we observe that binding to CB7 causes a small blue shift of 6 nm (17 meV), which was not predicted by our calculations. As suggested by Kostjukova *et al*. [37], adding a single water molecule to form a hydrogen bond with the nitrogen atom of the central ring of MeB (Figure 1(c)) in our calculations red-shifts the absorption spectrum of MeB in solution by 56 meV to 2.08 eV (Figure S7). Because such water molecule is always nearby in QM/MM MD simulations of MeB in water, but not in QM/MM MD simulations of the MeB–CB7 complex in water (Figure S11, SI), we speculate that the blue-shift of the spectrum is due to the loss of this hydrogen bond when the MeB binds to CB7.

Finally, we considered the possibility that two MeB molecules bind inside a single CB7. Although we could optimize a geometry in which two MeB molecules bind to the same CB7 (Figure S8, SI), classical MD simulations at the CHARMM36 force field level of this configuration in explicit water, suggest that a complex between CB7 and the dimer is thermodynamically unfavorable, as one of the MeB molecules rapidly exits the CB7 barrel. These findings indicate that CB7 accommodates at most one MeB molecule, an essential requirement for achieving single-molecule strong coupling in the NPoM nanocavity [24].

**Figure 3:**
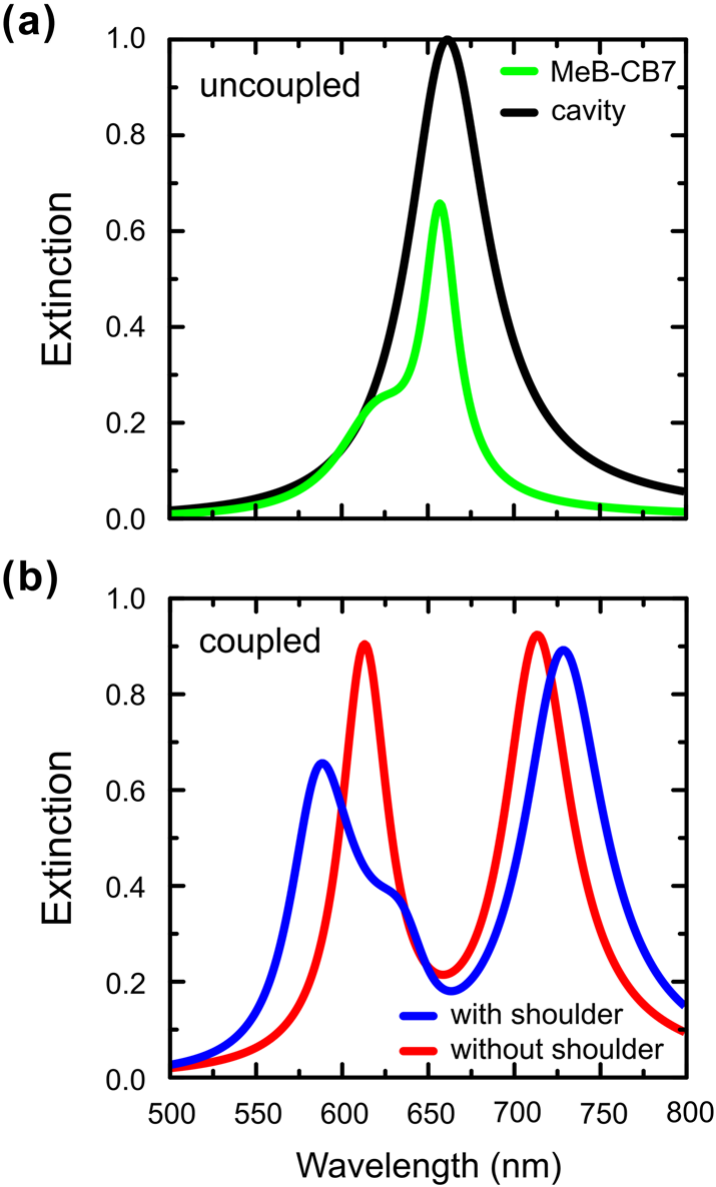
Simulated extinction spectra of a nanoparticle-on-mirror cavity containing a methylene blue – cucurbit[7]uril complex modeled with (blue) and without (red) resolved vibronic structure. Panel a shows the uncoupled spectra of the nanocavity mode (black) and MeB-CB7 complex (green), while panel b shows the spectra in the strong coupling regime.

The vibronic transition responsible for the absorption shoulder in MeB lies energetically close to the 0 → 0 line and can therefore also interact with the plasmonic mode of the NPoM cavity. To evaluate its impact on the strong coupling response, we simulated scattering spectra within the classical input/output formalism, representing the cavity mode and molecular transitions as Lorentzians (see SI), using either a single molecular transition (0 → 0 only) or two transitions (0 → 0 plus vibronic shoulder). Without the shoulder, the spectrum, shown in Figure 3, exhibits the symmetric Rabi splitting expected for a single two-level system, with comparable upper and lower polariton (UP/LP) intensities. Inclusion of the vibronic transition breaks this symmetry, resulting in a weaker LP and a more intense UP, as well as a non-Lorentzian LP lineshape with a weak high-energy shoulder, in qualitative agreement with experiment [24]. Additionally, the Rabi splitting increases when the vibronic transition is included, reflecting the collective coupling of both molecular transitions to the cavity mode. These results suggest that even a modest vibronic absorption can measurably influence polariton lineshapes and apparent coupling strength in single-molecule plasmonic cavities.

## 4 Conclusions

In summary, our TDDFT results confirm that the shoulder in the MeB absorption spectrum arises from a combination of vibronic progression in the monomer and excitonic absorption of the dimer [32], [34]. Upon binding to CB7, this feature decreases but does not disappear, in agreement with our experiments and with other published spectra [31], [70], [71], though inconsistent with the spectrum reported in Chikkaraddy *et al*. [24]. Despite the systematic overestimation of excitation energies by TDDFT [57], the calculated line shapes reproduce experiment well. This convergence highlights the robustness of our model and its suitability for future atomistic simulations of NPoM systems [72].

## Supplementary Material

Supplementary Material Details

Supplementary Material Details

Supplementary Material Details
