# Supplementary material for: Disentangling the absorption lineshape of methylene blue for nanocavity strong coupling
Source: Nanophotonics. 2025 Nov 25;14(27):4993–5001. doi: 10.1515/nanoph-2025-0474 (PMC12717915; doi:10.1515/nanoph-2025-0474)
Supplement: Supplementary file 1 — Supplementary Material Details [file j_nanoph-2025-0474_suppl_001.pdf]

Supporting information

for

Disentangling the Absorption Lineshape of Methylene Blue for Nanocavity Strong Coupling

Santiago A. Gomez,[†] Emmi Pohjolainen,[†] Dmitry Morozov,[†] Ville Tiainen,[‡] J. Jussi Toppari,[‡] and Gerrit Groenhof[*,†]

† *Nanoscience Center and Department of Chemistry, University of Jyväskylä, P.O. Box 35, 40014 Jyväskylä, Finland.*

‡ *Nanoscience Center and Department of Physics, University of Jyväskylä, P.O. Box 35, 40014 Jyväskylä, Finland.*

E-mail: gerrit.x.groenhof@jyu.fi

Contents

Theoretical Backgound 3

Methylene-Blue Spectrum 6

Methylene-Blue dimerization and Insertion in CB7 9

QM/MM molecular dynamics simulations 14

Binding free energy for the MeB complex with CB7 16

MeB–CB7 optical absorption measurements 18

Effect of vibronic shoulder on polariton lineshape 20

References 21

Theoretical Backgound

During a photoexcitation process, molecules in the ground electronic state (S_0) absorb photons. The excess of energy supplied by the photon causes the molecules to be promoted to a higher energy electronic level (S_1), which is called the excited state.

At the same time, in a polyatomic molecule, the collective motion of the atoms give rise to vibrations that can be described in terms of normal modes. For a molecule with N atoms, there are $3N-6$ (or $3N-5$ in the case of linear molecules) normal modes. Each of these vibrations has a characteristic energy that give rise to vibrational states ($v = \nu$). Every electronic state contains different vibrational states.

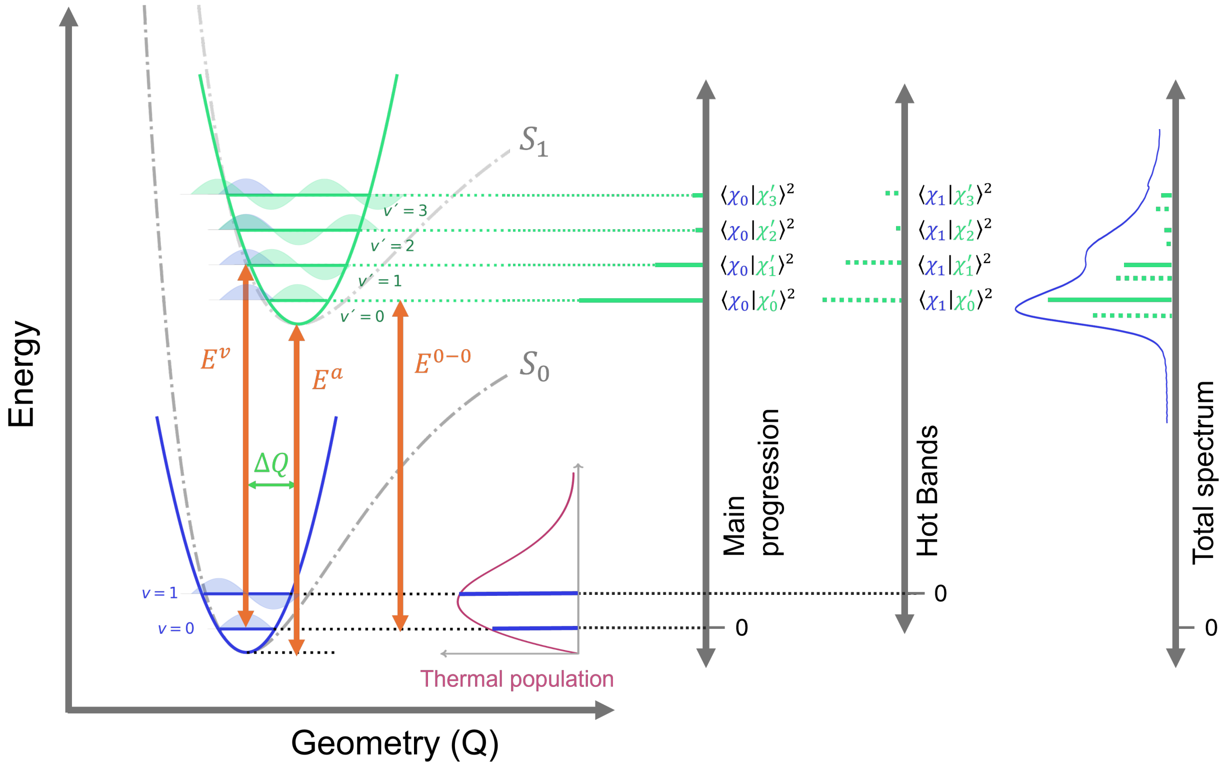

Figure S1: Potential energy diagram depicting a $S_0 \rightarrow S_1$ electronic transition. The potential energy surfaces of ground (S_0) and excited (S_1) electronic states are depicted as Morse potentials. Vibrational electronic states ($v = \nu$ and $v' = \nu$) are represented as stationary waves. Vertical transition energy (E^v), adiabatic energy (E^a) and $0 \rightarrow 0$ transition energy (E^{0-0}) are also shown. The total spectrum is the result of all possible transition between vibronic (vibrational plus electronic) levels with an intensity given by the Franck-Condon factors $\langle\chi_\nu|\chi'_\nu\rangle^2$. Figure adapted from Gozem and Krylov.[1]

The electronic transition can occur between any of the vibrational states in the ground state and any of the vibrational states in the excited state, depending on the energy supplied by the absorbed photon, producing a spectrum of bands rather than lines.

The Franck-Condon principle describes why some bands are more intense than others. Figure S1 shows the electronic states S_0 and S_1 as a function of the collective positions of the atoms in the molecule (Q). Because electronic transitions occur faster than the movements of atomic nuclei and Q does not change, a vertical transition (E^v) is observed in the potential energy diagram. The intensity (or probability) of a transition between two vibrational levels $v = \nu$ in the ground state and $v' = \nu$ in the excited state depends on the overlap between the vibrational wavefunctions, the so called Franck-Condon factor:[2]

$$FC_{v,v'} = \left| \int \chi_\nu^*(Q)\chi_\nu'(Q)dQ \right|^2 = \langle \chi_\nu | \chi_\nu' \rangle^2$$

A large overlap implies a more intense transition. At low temperatures (0 K), only the fundamental vibrational level $v = 0$ is populated in S_0, which means that only transitions $v = 0 \rightarrow v' = \nu$ will occur, resulting in the main progression bands. As the temperature increases, the population of the different vibrational levels in the ground electronic state follows the Boltzmann distribution (depicted as Thermal population in Figure S1), allowing transitions from vibrational levels $v = \nu$ with $\nu \neq 0$, and leading the hot bands. The convolution of all these bands yields the absorption spectra of a molecule.

In order to theoretically obtain an absorption spectrum, it is necessary to know the potential energy surface (the energy of all vibrational states) of each electronic level.[3] To do this, it is necessary to solve the Schrödinger equation for each vibrational state, which is extremely expensive. Instead, one can use the harmonic approximation, in which the potential energy surface (PES) is modelled as a harmonic oscillator.[3,4] In Figure S1, the original shape of the PES is represented as a Morse potential for each electronic state S_n and the approximation is shown as a parabola describing the harmonic potential.

Under this approximation, the PES of S_0 is expressed around the equilibrium geometry of the ground state in the matrix form:

$$V(\mathbf{Q}) = \frac{1}{2}\mathbf{Q}^T\Omega^2\mathbf{Q}$$

Were the normal frequency modes are contained in the diagonal matrix Ω and the vector $\mathbf{Q}$ list the N associated normal coordinates. In the Adiabatic Hessian (AH), the model used in this work, the PES of the excited state S_1 is also expressed around its equilibrium geometry in matrix form:

$$V(\mathbf{Q}') = E^a + \frac{1}{2}\mathbf{Q}'^{\,T}\Omega^2\mathbf{Q}'$$

With E^a the adiabatic energy (difference between S_1 and S_0 energy minima). The normal coordinates of the ground and excited states are related by the equation:

$$\mathbf{Q}' = \mathbf{JQ} + \mathbf{K}$$

With $\mathbf{K}$ the displacement vector of the equilibrium position and $\mathbf{J}$ the rotation matrix (also known as Duschinsky matrix), which accounts for the overlap between $\mathbf{Q}$ and $\mathbf{Q}'$. Finally, the computation of the spectral lineshape is obtained (in the time independent TI formalism, *i.e*, Fermi's Golden Rule) as a sum of all state-to-state $|\chi_v\rangle \rightarrow |\chi'_{v'}\rangle$ vibronic transitions. For a one photon process:[3,4]

$$L(\omega) = \sum_{v,v'} \rho_v(T)|\langle v|\mu^{S_0\rightarrow S_1}|v'\rangle|^2\delta(E^v + \hbar\omega)$$

Here, $\rho_v(T)$ is the Boltzmann population of the $v = \nu$ state at temperature T and $\mu^{S_0\rightarrow S_1}$ is the electronic transition moment $\langle S_0|\hat{\boldsymbol{\mu}}|S_1\rangle$ with $\hat{\boldsymbol{\mu}}$ the electronic transition dipole moment operator.

Methylene-Blue Spectrum

To investigate the effect of temperature on the absorption spectrum, we computed the vibronic spectra at 0 and 300 K, and show the results in Figure S2. The lack of a significant difference between these spectra suggests that the spectrum at 300 K is dominated by the $v = 0 \rightarrow v' = \nu$ transitions.

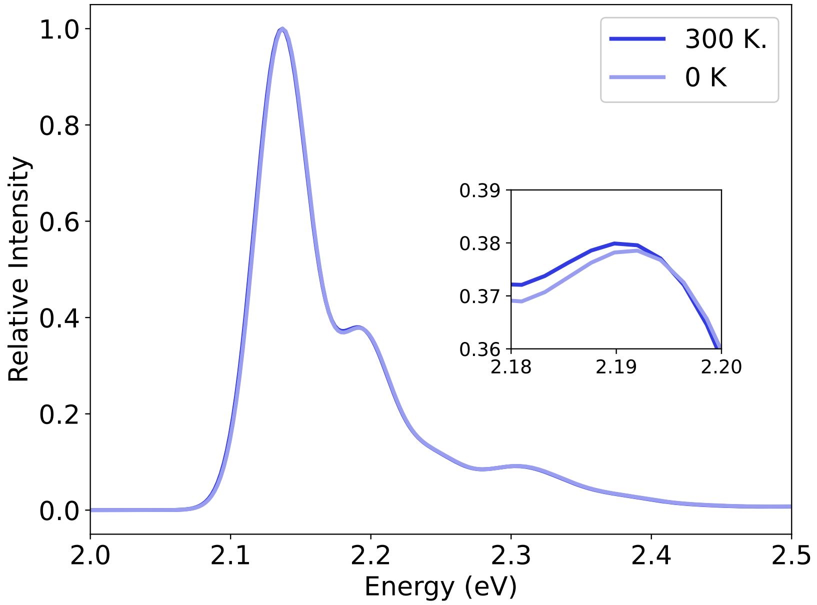

Figure S2: Simulated spectra of Methylene-Blue at 0 and 300 K

The reorganization energy for the photoexcitation process is defined as the difference between the vertical and adiabatic excitation energies:

$$E_{\text{abs}}^{\text{reorg}} = E^v - E^a$$

Alternatively, the reorganization energy can be calculated as the sum over all normal modes frequencies (ω_j) weighted by their Huang-Rhys factors (S_j):

$$E_{\text{abs}}^{\text{reorg}} = \sum_j \hbar \omega_j S_j$$

A small reorganization energy implies a small displacement between electronic states minima. Under such conditions, the Harmonic approximation is valid. To test the validity of of this approximation for our systems, we compare the reorganization energies obtained with both approaches in Table S1. For all systems considered in this work, the differences are sufficiently small to justify the use of the Harmonic approximation.

Table S1: **Photoexcitation reorganization energies for each studied system in eV**

	MeB	MeB_2 (A)	MeB_2 (B)	MeB−CB7
$E^v - E^a$	0.0412	0.0253	0.0448	0.0533
$\sum_i \hbar\omega_i S_i$	0.0419	0.0257	0.0587	0.0582

The vibronic absorption spectrum of MeB was simulated using FCClasses version 3.0.[5] The resulting spectrum is shown in Figure S3 as well as normal modes involved in the vibronic excitations responsible for the shoulder.

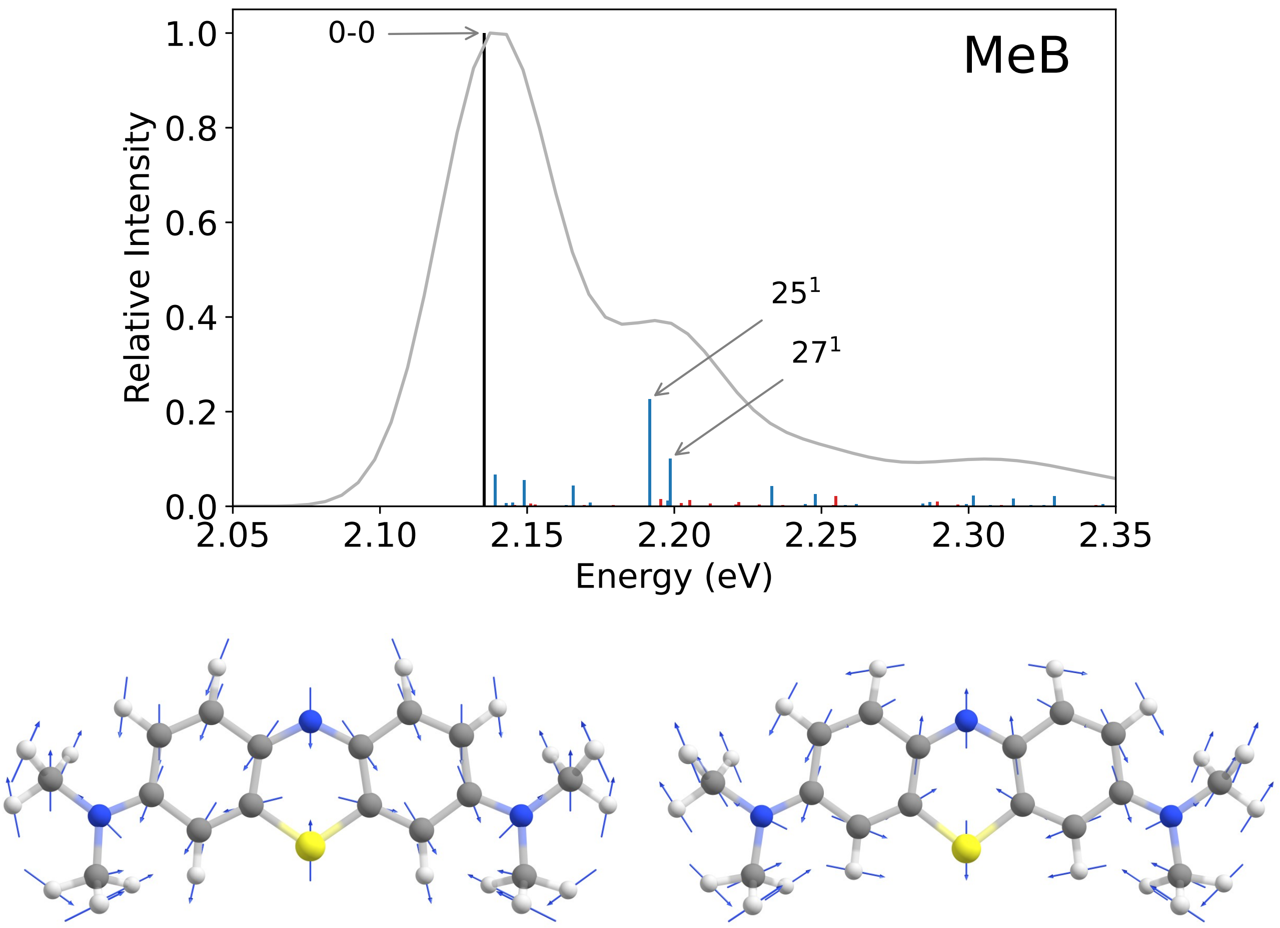

Mode 25 (454 cm^{-1}) Mode 27 (510 cm^{-1})

Figure S3: Vibronic Spectra of Methylene Blue monomer in aqueous solution at 300 K calculated at the TD-CAM-B3LYP/6-31G(d) level of theory. The picture in the **bottom**, shows the calculated normal vibrational modes (and their wave numbers) responsible for the shoulder in the absorption spectrum.

Methylene-Blue dimerization and Insertion in CB7

To compute the complexation and dimerization energies, we perform a CAM-B3LYP/6-31G(d) structure optimization in implicit PCM solvent for the whole systems and each constituent molecules, and subtract the latter from the former. The basisset superposition error (BSSE) was calculated in gas phase at the same level of theory and using counterpoise corrections implemented in Gaussian16.[6] The obtained values are listed in Table S2:

Table S2: **Complexation Energy:** Energy in kcal mol^{-1} of MeB dimerization and MeB-CB7 complex formation calculated at CAM-B3LYP/6-31G(d) level of theory and corrected for the Basis Set Superposition Error (BSSE).

System	$\Delta E_{complex}$	BSSE Energy	$\Delta E_{complex}$ Corrected
MeB−CB7	-48.26	14.23	-34.03
MeB_2 Parallel (A)	-18.88	4.83	-14.06
MeB_2 Anti-parallel (B)	-19.08	4.75	-14.33

The Vibronic Spectrum of MeB−CB7 complex is shown in Figure S4. The line shape of the complex is to the line shape of MeB in solution (Figure S3) and the vibrational modes involved in the vibronic transitions that constitute the shoulder are practically identical (Table S3)

Table S3: **Vibrational modes:** Transition energy (in eV) and frequency (in cm^{-1}) of normal modes producing the shoulder in the absorption spectra of MeB in water and in CB7.

System	Transition	ΔE transition	Frequency
MeB	$0 \rightarrow 25^1$	2.19	454
	$0 \rightarrow 27^1$	2.20	510
MeB−CB7	$0 \rightarrow 107^1$	2.18	459
	$0 \rightarrow 115^1$	2.19	515

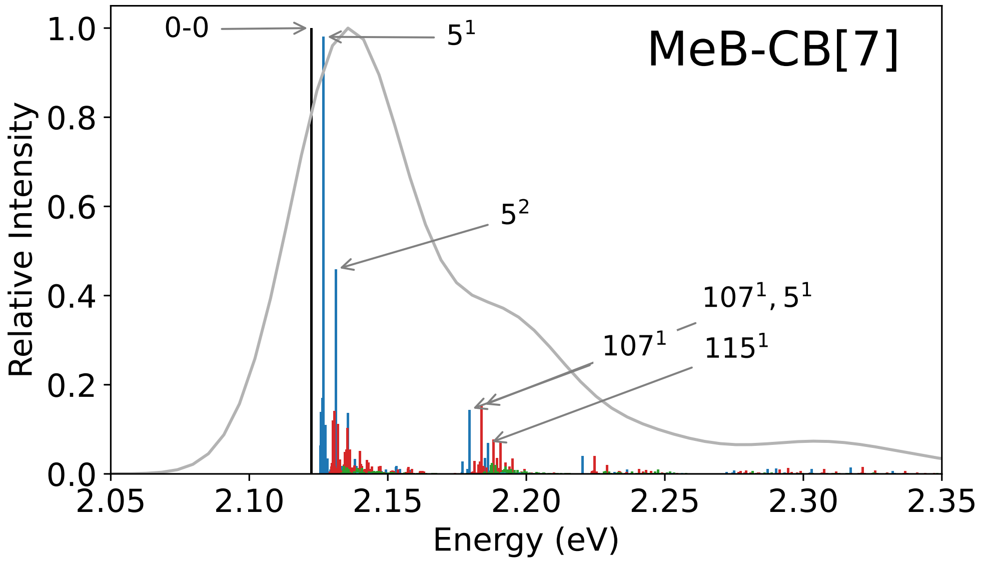

Figure S4: Vibronic Spectra of Methylene Blue monomer encapsulated in Cucurbit[7]uril at 300 K, calculated at TD-CAM-B3LYP/6-31G(d) level of approximation. Vibronic transitions producing the broadening of main peak and shoulder are highlighted.

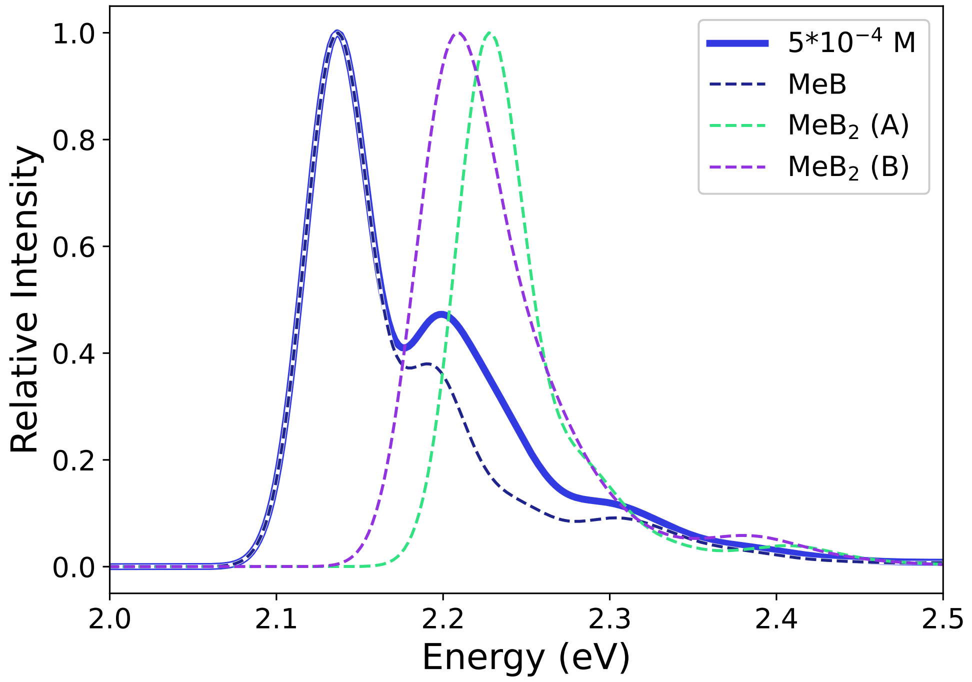

Figure S5: Calculated spectrum for an aqueous solution of Methylene-Blue at $5{\cdot}10^{-4}$ M. Absorption spectra of MeB monomer and dimers are also shown. Here, the dimer absorption is red-shifted by 0.02 eV. The contribution of dimer absorption increases the height of the shoulder, bringing the calculated spectral line shapes in closer agreement with experiment (Figure S6b)

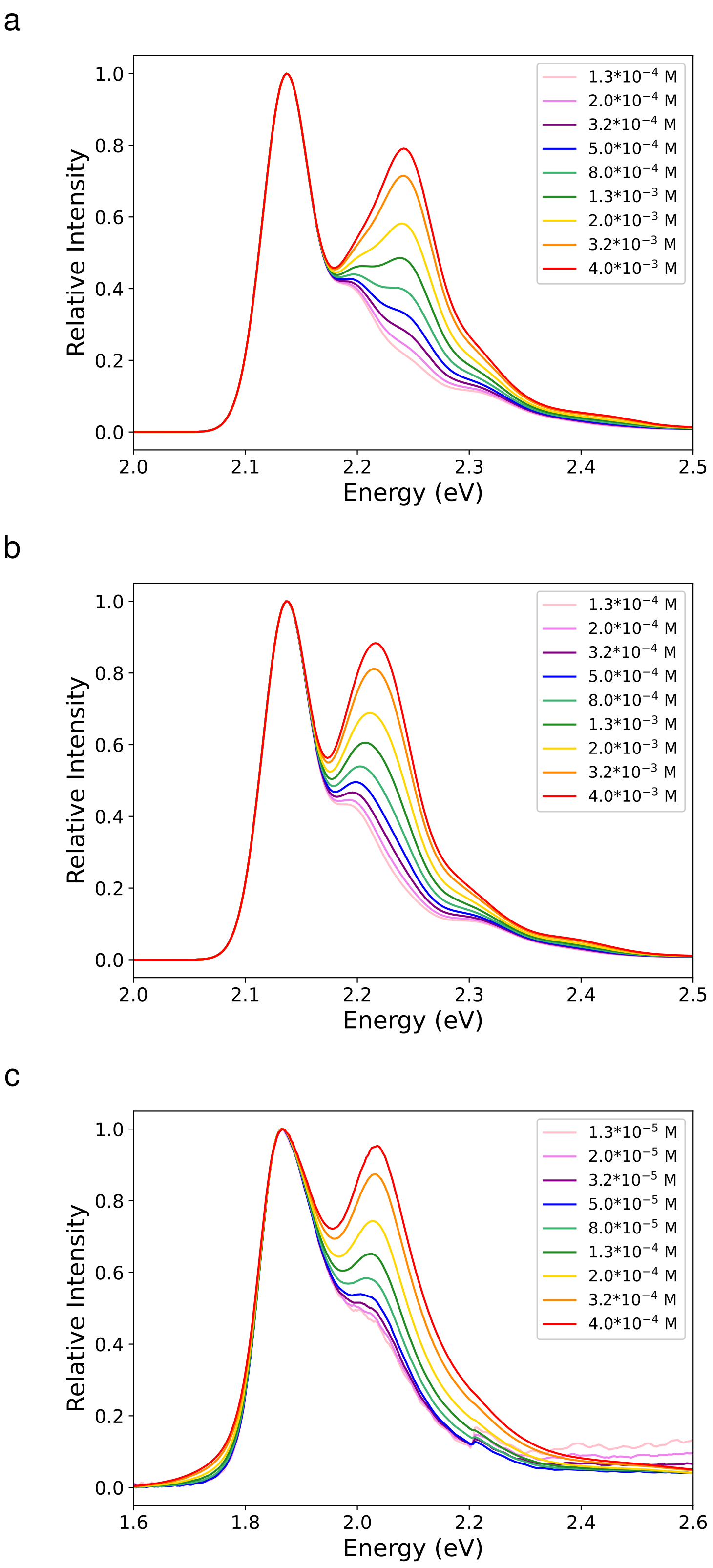

Figure S6: Calculated spectra without (**a**) and with (**b**) a dimers peak red shift of 0.02 eV, and measured (**c**) absorption spectra of Methylene Blue solutions at various concentrations. The spectral height of the shoulder increases with the concentration, suggesting that the shoulder is due to the dimer, in line with previous findings.[7,8] The minor discontinuities around 2.2 eV in the experimental spectrum arise from automatic long-pass filter switching in the spectrometer light path and are not intrinsic to the sample.

Including an explicit water molecule forming a hydrogen bond with the nitrogen atom of the central ring in MeB, leads to red shift of the absorption spectra in our TDDFT calculations.

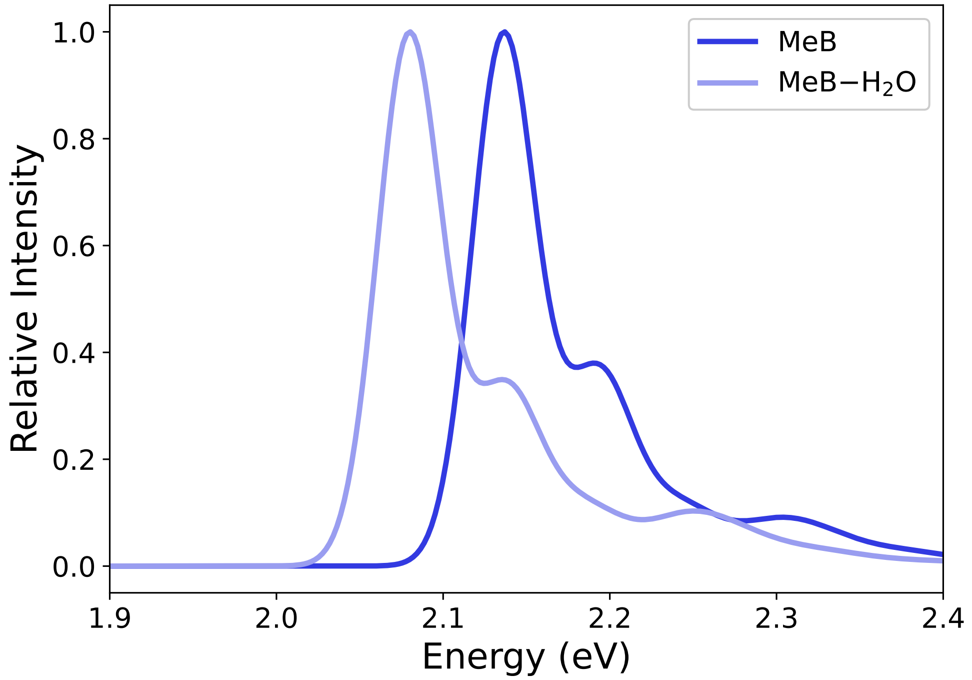

Figure S7: Calculated absorption spectra for Methylene-Blue with and without an water molecule forming a hydrogen bond with the nitrogen atom in the central ring of MeB. The rest of the water is modeled implicitly, using the polarizable continuum model (PCM).[9]

After the insertion of two Methylene-Blue molecules in CB7, we optimized the complex at the CAMB3LYP/6-31G(d) level of theory with dispersion corrections[10] in implicit water (PCM).[9] The optimized geometry of the complex is shown in Figure S8. Next, we performed a 100 ns molecular dynamics simulation of the complex in water, using the CHARMM36 forcefield,[11] in combination with the TIP3P model for the water molecules.[12] During the first 100 ps one MeB left the CB7, while the other MeB remained inside for the rest of the simulation. Based on these observations, we concluded that the encapsulating a dimer is thermodynamically unfavorable. We therefore did not attempt to compute the free energy profile.

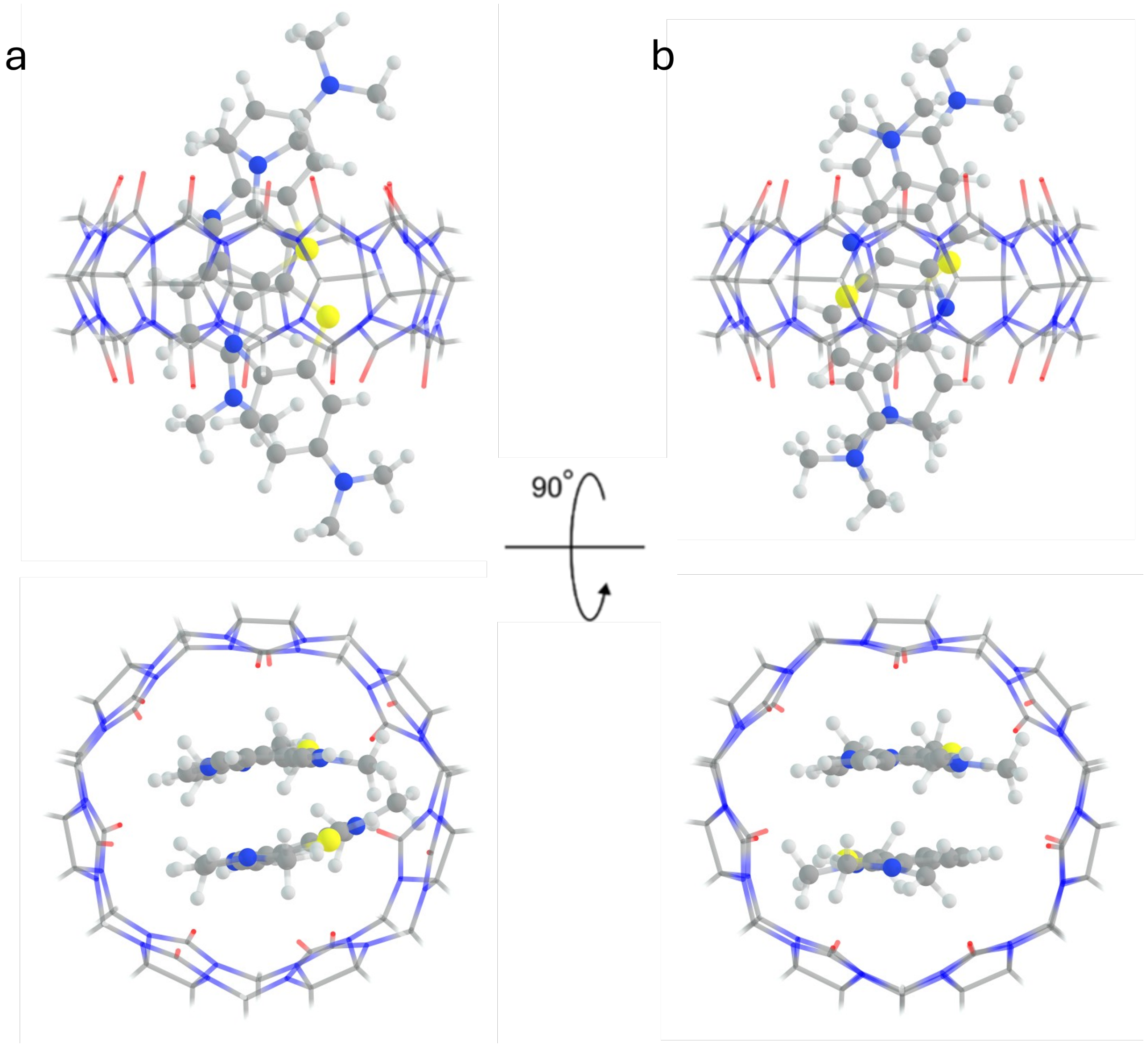

Figure S8: Front (**top**) and top (**bottom**) views of Cucurbit[7]uril in complex with Methylene-Blue dimer with (**a**) parallel and (**b**) anti-parallel conformations.

QM/MM molecular dynamics simulations

In Figure S9, we show the energies of the four lowest energy singlet states along a CAMB3LYP/6-31G(d)//CHARMM36 QM/MM MD trajectory of Methylene Blue in water (TIP3P). The energies of the ground and excited states were evaluated at the TD-CAMB3LYP/6-31G(d) and CIS(D)/6-31G(d) levels of theory. Although the energies are overestimated at the TDDFT level, the curvatures are similar.

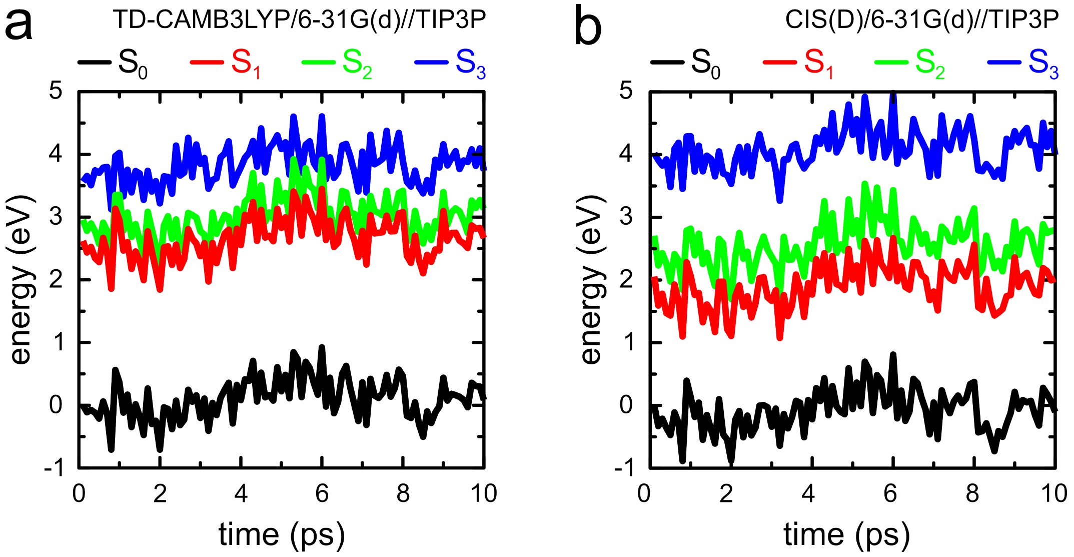

Figure S9: Energies of the four lowest energy singlet states along a classical QM/MM molecular dynamics trajectory, evaluated at the (**a**) TD-CAMB3LYP level of time-dependent DFT theory and the (**b**) CIS(D) level of ab initio theory.

In Figure S10, we show the energy gap between the S_2 and S_1 states along a CAMB3LYP/6-31G(d)//CHARMM36 trajectory of Methylene Blue in water (TIP3P). The gaps were computed at both the TD-CAMB3LYP/6-31G(d)//CHARMM36 and CIS(D)/6-31G(d)//CHARMM36 levels of theory.

In Figure S11, we show the minimum distance between the Nitrogen atom in the central ring of Methylene Blue and water molecules as a function of simulation time in a QM/MM trajectory of Methylene Blue in water and of the Cucurbit[7]uril–Methylene Blue host–guest complex in water. Cucurbit[7]uril and water were modeled with the CHARM36 and TIP3P force fields, respectively. Methylene Blue was described at the CAMB3LYP/6-31G(d) level of DFT. A distance around 0.2 nm

indicates the presence of a hydrogen bond.

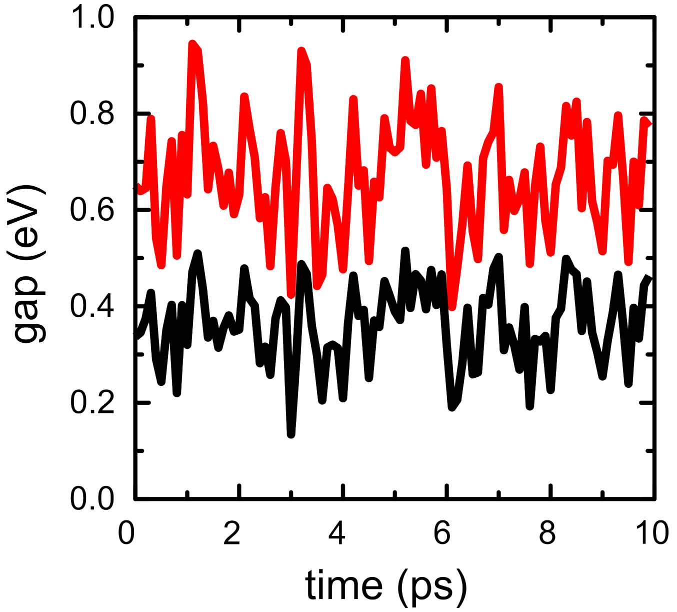

Figure S10: Energy gap between the S_2 and S_1 states along a classical QM/MM molecular dynamics trajectory, computed at the TD-CAMB3LYP level of time-dependent DFT theory (black) and the CIS(D) level of *ab initio* theory (red).

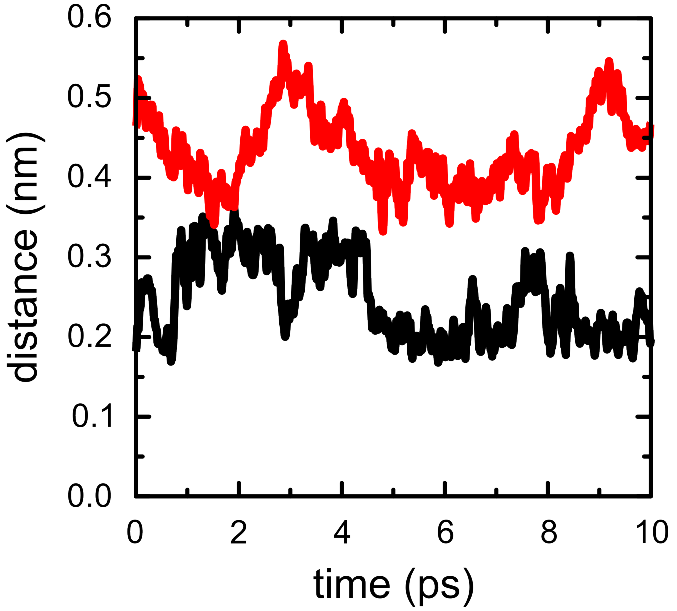

Figure S11: Minimum distance between the nitrogen atom of the central ring of Methylene Blue and the water molecules in a simulation of Methylene Blue (black) and Cucurbit[7]uril–Methylene Blue host–guest complex (red) in water.

Binding free energy for the MeB complex with CB7

The complexation free energy was computed by means of enhanced sampling molecular dynamics simulations along a collective variable. As a collective variable, we used the distance between the centers of mass of MeB and CB7 (Figure S12A), and used the AWH method to enhance the sampling along this reaction coordinate.[13] The inital structure of the MeB–CB7 complex was constructed and equilibrated as outline in the Methods section of main text. This equilibration run was continued for another 100 nanoseconds of equilibration at constant pressure[14] and temperature,[15] with restraints to keep the principal axes of the MeB–CB7 complex aligned along the x-axis of the simulation box. After equilibration, we performed a 500 ns accelerated weight histogram (AWH) simulation at constant temperature using the v-rescale thermostat ($T_{\text{ref}} = 300$ K; $\tau_T = 100$ fs)[15] and pressure using the C-rescale barostat ($p_{\text{ref}} = 1$ bar; $\tau_p = 2$ ps)[14] with a timestep of 1 fs. Sampling was done every 10 steps, and the biasing potential was updated every 100 steps. The interval for the reaction coordinate sampling was set to 0 – 2 nm.

The free-energy profile, obtained by convolution of the sampling data, is presented in Figure S12B. From this profile, we estimate that the free energy associated with binding MeB to CB7 is is approximately 61 kJ·mol^{-1} (14.5 kcal·mol^{-1}). Notably, the free-energy profile has two minima, associated with two distinct binding modes of MeB at distances of 0.16 nm and 0.53 nm along the reaction coordinate. The global minimum, which represents the most stable binding conformation, is shown in Figure S12C. The second minimum, depicted in Figure S12D, corresponds to an additional (meta)stable configuration 6.3 kJ·mol^{-1} (1.5 kcal·mol^{-1}) higher than the global minimum. These findings suggest that there are multiple binding modes, with similar stability. Whether both binding modes are relevant for the spectra, or for strong coupling is the focus of an ongoing investigation.

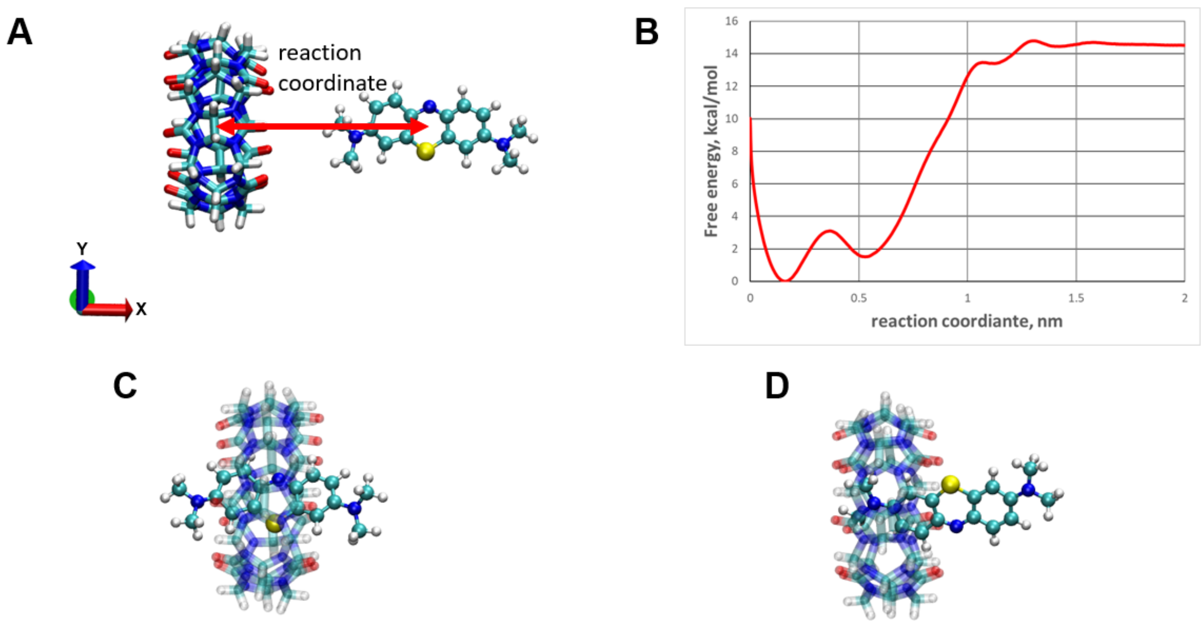

Figure S12: Results of the enhanced sampling Molecular Dynamics simulation with the adaptive weighted histogram method.[13] Panel A shows the initial alignment between MeB and CB7, and the reaction coordinate along which the free energy profile was computed. Panel B shows a plot of the free energy profile obtained from the AWH MD simulations. Panels C and D show representative structures of the global and local free energy minima of the host–guest complexes, respectively.

MeB–CB7 optical absorption measurements

Starting with a solution of Methylene Blue at $5{\cdot}10^{-5}$ M, we measured the absorption spectra while adding CB7 with different concentration ratios of MeB:CB7. All measured test solutions as well as stock solutions to mix them, are listed in Table S4. As seen in Figure S13, using an excess of MeB with respect to CB7, causes a rise in the shoulder. This observation suggests there is competition between CB7 binding and MeB oligomerization. However, the shoulder remains visible for excess CB7, suggesting that part of the shoulder is associated with the monomer. Based on the results of our computations, we attribute that part to vibronic progression. The measured spectra are available as supporting information.

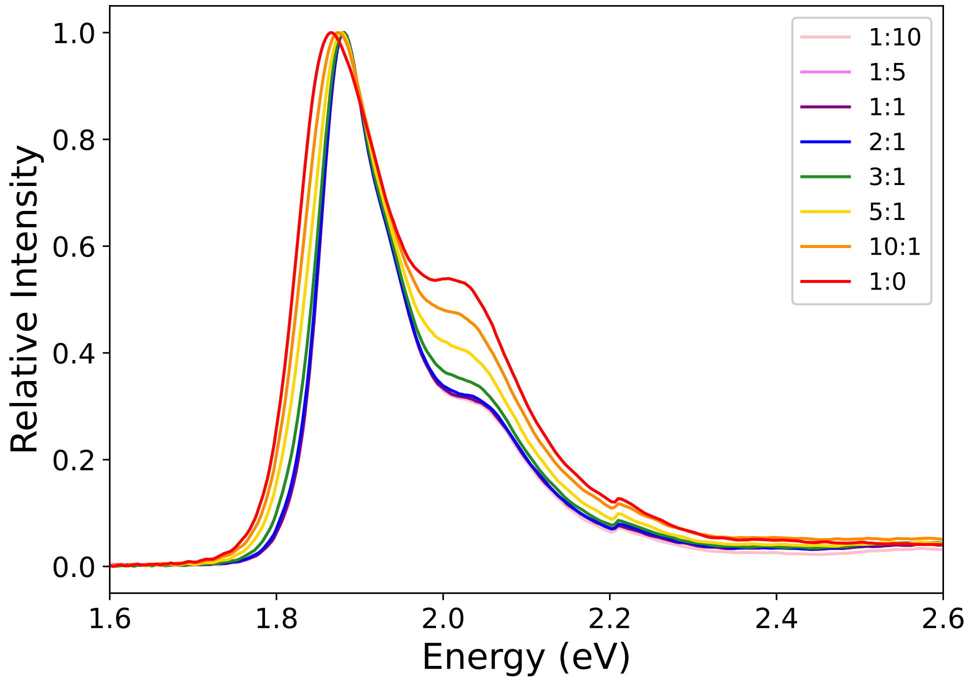

Figure S13: Experimental absorption spectra for MeB solution at $5{\cdot}10^{-5}$ M in combination with CB7 at different MeB:CB7 concentration ratios. Increasing the concentration of MeB in respect to CB7 increases the shoulder height due to enhanced dimerization.

Table S4: Stock- and test solution recipes.

**Stock Solutions**					
**Compound**	**CAS Code**	**Molar Mass**	**Amount**	**Solvent Volume**	**Concentration**
		**g mol^{-1}**	**mg**	**mL**	**mM**
MeB	61-73-4	319.85	4.8	1.5	10.0
CB7	259886-50-5	1163.0	17.45	1.5	10.0
**Test Solutions (Fixed MB = 400.5 μM)**					
**MeB:CB7 Ratio**	**MeB Stock**	**CB7 Stock**	**Water**	**[MeB]**	**[CB7]**
	**μL**	**μL**	**μL**	**μL**	**μL**
1:0	40.05	0	959.95	400.5	0
1:1	40.05	40.05	919.90	400.5	400.5
1:5	40.05	200.25	759.70	400.5	2002.5
1:10	40.05	400.5	559.45	400.5	4005.0
2:1	40.05	20.025	939.925	400.5	200.25
3:1	40.05	13.35	946.60	400.5	133.5
5:1	40.05	8.01	951.94	400.5	80.1
10:1	40.05	4.005	955.94	400.5	40.05

Effect of vibronic shoulder on polariton lineshape

To provide an estimate for the impact of the vibronic shoulder of MeB on the extinction spectrum when strongly coupled to the NPoM nanocavity, we use the classical input/output formalism to compute a scattering spectrum:

$$s_{\text{out}}(\omega) = \left(1 - \frac{\kappa_{\text{ext}}}{(\omega - \omega_{\text{p}}) + i\kappa_{\text{p}}/2 + \Sigma(\omega)}\right) s_{\text{in}}(\omega) \tag{1}$$

Here, $s_{\text{in}}(\omega)$ and $s_{\text{out}}(\omega)$ are the in- and output, respectively; ω_p is the resonance frequency of the plasmonic cavity; κ_{p} the cavity loss rate; κ_{ext} the coupling rate to the collection channel; and $\Sigma(\omega)$ the molecular self-energy, defined as

$$\Sigma(\omega) = \sum_j \frac{g_j^2}{(\omega - \omega_j) + i\gamma_j/2} \tag{2}$$

with g_j the cavity-molecule coupling rate for transition j with frequency ω_j and linewidth γ_j. The parameters for this model were obtained from fitting Lorentzians to our experimental spectra of the MeB-CB7 complex (Figure 1d), and the experimental scattering spectra of individual NPoMs in Figure 3a of Chikkarreddy *et al.*[16] All parameters are listed in Table S5. The spectrum plotted in Figure 3 of the main text is the extinction, calculated as $\text{Ext}(\omega) = 1 - |s_{\text{out}}(\omega)/s_{\text{in}}(\omega)|^2$.

Table S5: **Parameters for input/output model.**

parameter	value (eV)
ω_1	1.90
γ_1	0.07
g_1	0.143
ω_2	2.00
γ_2	0.19
g_2	0.139
ω_{p}	1.88
κ_{p}	0.158
κ_{ext}	0.079

References

(1) Gozem, S.; Krylov, A. I. The ezSpectra suite: An easy-to-use toolkit for spectroscopy modeling. *Wiley Interdisciplinary Reviews: Computational Molecular Science* **2022**, *12*, e1546.

(2) Bernath, P. F. *Spectra of atoms and molecules*; Oxford university press, 2020.

(3) Ferrer, F. J. A.; Santoro, F. Comparison of vertical and adiabatic harmonic approaches for the calculation of the vibrational structure of electronic spectra. *Physical Chemistry Chemical Physics* **2012**, *14*, 13549–13563.

(4) Santoro, F.; Jacquemin, D. Going beyond the vertical approximation with time-dependent density functional theory. *Wiley Interdisciplinary Reviews: Computational Molecular Science* **2016**, *6*, 460–486.

(5) Cerezo, J.; Santoro, F. FCclasses3: Vibrationally-resolved spectra simulated at the edge of the harmonic approximation. *J. Comp. Chem.* **2023**, *44*, 626–643.

(6) Frisch, M. J.; Trucks, G. W.; Schlegel, H. B.; Scuseria, G. E.; Robb, M. A.; Cheeseman, J. R.; Scalmani, G.; Barone, V.; Petersson, G. A.; Nakatsuji, H.; Li, X.; Caricato, M.; Marenich, A. V.; Bloino, J.; Janesko, B. G.; Gomperts, R.; Mennucci, B.; Hratchian, H. P.; Ortiz, J. V.; Izmaylov, A. F.; Sonnenberg, J. L.; Williams-Young, D.; Ding, F.; Lipparini, F.; Egidi, F.; Goings, J.; Peng, B.; Petrone, A.; Henderson, T.; Ranasinghe, D.; Zakrzewski, V. G.; Gao, J.; Rega, N.; Zheng, G.; Liang, W.; Hada, M.; Ehara, M.; Toyota, K.; Fukuda, R.; Hasegawa, J.; Ishida, M.; Nakajima, T.; Honda, Y.; Kitao, O.; Nakai, H.; Vreven, T.; Throssell, K.; Montgomery, J. A., Jr.; Peralta, J. E.; Ogliaro, F.; Bearpark, M. J.; Heyd, J. J.; Brothers, E. N.; Kudin, K. N.; Staroverov, V. N.; Keith, T. A.; Kobayashi, R.; Normand, J.; Raghavachari, K.; Rendell, A. P.; Burant, J. C.; Iyengar, S. S.; Tomasi, J.; Cossi, M.; Millam, J. M.; Klene, M.; Adamo, C.; Cammi, R.; Ochterski, J. W.; Martin, R. L.; Morokuma, K.; Farkas, O.; Foresman, J. B.; Fox, D. J. Gaussian~16 Revision C.01. 2016; Gaussian Inc. Wallingford CT.

(7) Florence, N.; Naorem, H. Dimerization of methylene blue in aqueous and mixed aqueous organic solvent: A spectroscopic study. *J. Mol. Liq.* **2014**, *198*, 255–258.

(8) Dean, J. C.; Oblinsky, D. G.; Rather, S. R.; Scholes, G. D. Methylene Blue Exciton States Steer Nonradiative Relaxation: Ultrafast Spectroscopy of Methylene Blue Dimer. *J. Phys. Chem. B* **2016**, *120*, 440–454.

(9) Tomasi, J.; Mennucci, B.; Cancès, E. The IEF version of the PCM solvation method: An overview of a new method addressed to study molecular solutes at the QM ab initio level. *J. Mol. Struct.(Theochem)* **1999**, *464*, 211–26.

(10) Grimme, S.; Antony, J.; Ehrlich, S.; Krieg, H. A consistent and accurate ab initio parametrization of density functional dispersion correction (DFT-D) for the 94 elements H-Pu. *The Journal of Chemical Physics* **2010**, *132*, 154104.

(11) Huang, J.; MacKerell Jr, A. D. CHARMM36 all-atom additive protein force field: Validation based on comparison to NMR data. *Journal of computational chemistry* **2013**, *34*, 2135–2145.

(12) Jorgensen, W. L.; Chandrasekhar, J.; Madura, J. D.; Impey, R. W.; Klein, M. L. Comparison of simple potential functions for simulatin liquid water. *J. Chem. Phys.* **1983**, *79*, 926–935.

(13) Lindahl, V.; Lidmar, J.; Hess, B. Accelerated weight histogram method for exploring free energy landscapes. *J. Chem. Phys.* **2014**, *141*, 044110.

(14) Bernetti, M.; Bussi, G. Pressure control using stochastic cell rescaling. *The Journal of Chemical Physics* **2020**, *153*, 114107.

(15) Bussi, G.; Donadio, D.; Parrinello, M. Canonical sampling through velocity rescaling. *J. Chem. Phys.* **2007**, *126*, 014101.

(16) Chikkaraddy, R.; de Nijs, B.; Benz, F.; Barrow, S. J.; Scherman, O. A.; Rosta, E.; Demetriadou, A.; Fox, P.; Hess, O.; Baumberg, J. J. Single-molecule strong coupling at room temperature in plasmonic nanocavities. *Nature* **2016**, *535*, 127–130.
